# MicroRNA-34a Inhibition Alleviates Lung Injury in Cecal Ligation and Puncture Induced Septic Mice

**DOI:** 10.3389/fimmu.2020.01829

**Published:** 2020-08-13

**Authors:** Song Chen, Renyu Ding, Ziwei Hu, Xiaohan Yin, Feng Xiao, Wei Zhang, Shijiao Yan, Chuanzhu Lv

**Affiliations:** ^1^Department of Trauma Intensive Care Unit, The First Affiliated Hospital of Hainan Medical University, Haikou, China; ^2^Key Laboratory of Emergency and Trauma of Ministry of Education, Hainan Medical University, Haikou, China; ^3^Intensive Care Unit, The First Affiliated Hospital of China Medical University, Shenyang, China; ^4^Department of Critical Care Medicine, The Central Hospital Affiliated to Shenyang Medical College, Shenyang, China; ^5^Department of Emergency Medicine, The Second Affiliated Hospital of Hainan Medical University, Haikou, China; ^6^Emergency and Trauma College, Hainan Medical University, Haikou, China; ^7^School of Public Health, Hainan Medical University, Haikou, China

**Keywords:** sepsis, lung injury, microRNA-34a, oxidative stress, pyroptosis, autophagy

## Abstract

Sepsis is the leading cause of death in intensive care units. MicroRNA-34a (miR-34a) is involved in sepsis progression, while its underlying mechanisms on sepsis-induced lung injury remain obscure. Oxidative stress, pyroptosis, and inhibition of autophagy can result in organ injury. MiR-34a has been reported to regulate oxidative stress and autophagy via inhibiting silent information regulator T1 (SIRT1) and autophagy gene 4B (ATG4B) signaling. This study aimed at identifying the function of miR-34a in oxidative stress, inflammation, pyroptosis, and autophagy in sepsis-induced lung injury. Male 8-week-old C57BL/6 mice were subjected to cecal ligation and puncture and treated with miR-34a antagomir/agomir. Survival (*n* = 10), histopathological changes (*n* = 6), and lung wet-to-dry ratio (*n* = 6) were recorded and assayed. Other detection (*n* = 6) was performed to investigate the level of oxidative stress, inflammation, pyroptosis, and autophagy in lung tissues. Results showed that miR-34a down-regulation ameliorated lung injury in septic mice as reflected by decreased lung injury scores (decrease from 3.00 ± 0.32 to 2.00 ± 0.32) and wet-to-dry ratio (0.36-fold decrease). MiR-34a down-regulation also decreased reactive oxygen species accumulation (0.36-fold decrease), and promoted superoxide dismutase activity and the expression of SIRT1 (1.24-fold increase), heme oxygenase-1 and nuclear factor erythroid 2 like 2 to inhibit oxidative stress in septic mice. Moreover, miR-34a down-regulation suppressed inflammatory response and pyroptosis in septic mice, as evidenced by decreased level of pro-inflammatory factors including tumor necrosis factor α, interleukin-6 (IL-6), IL-1β, and IL-18, activity of caspase-1 (0.51-fold decrease) and expression of nucleotide-binding domain and leucine-rich repeat protein-3 (0.48-fold decrease), apoptosis-associated speck-like protein containing a CARD, cleaved-caspase-1, and cleaved-gasdermin D (0.36-fold decrease), and increased level of anti-inflammatory factors IL-10. MiR-34a down-regulation also enhanced autophagy in septic mice as evidenced by more autolysosomes and elevated expressions of ATG4B (0.90-fold increase), beclin1, ATG9, and LC3 II/I. Among these experiments, miR-34a up-regulation showed opposite effects on oxidative stress, inflammatory response, pyroptosis, and autophagy in septic mice. Additionally, miR-34a could bind to the 3′-untranslated region of SIRT1 and ATG4B. In conclusion, our findings demonstrated that miR-34a was implicated in oxidative stress, inflammation, pyroptosis, and autophagy in the development of sepsis. MiR-34a inhibition had a potential to alleviate sepsis-induced lung injury.

## Introduction

Sepsis is a heterogeneous syndrome induced by unbalanced host response to infection which is characterized by organ dysfunction (1). Sepsis is one of the leading causes for hospitalized patients in non-coronary intensive care units (2). Despite therapeutic innovations, the incident case of sepsis remains ~48.9 million, and the mortality remains ~11.0 million worldwide (3). Sepsis represents a heavy economic and health care burden. Sepsis also severely affects the life quality of patients and those who survive sepsis usually suffer from multiple comorbidities (4). Gram-negative bacteria, gram-positive bacteria, and fungi are major causative organisms of sepsis which result in infections and disturbed homeostasis of immune system (5). The host response to invaded pathogen is disturbed by excessive inflammation and immune suppression, and will further lead to aberrant immune response which persists even after the treatment of infection (6). The constant unbalanced immune response triggers a series of organ dysfunctions, among which lung injury is most common in clinics (5). To date, the recommended treatment for sepsis includes source control, antibiotics, fluid resuscitation, vasoactive mediation as well as organ function support. Unfortunately, all the current managements of sepsis have their limitations and fail to efficiently lower incidence rate and mortality (7). Therefore, a better understanding of sepsis pathogenesis and discovering novel clinical therapies are of great significance to public health.

Approximately 80% of genetic elements are aberrantly expressed in patients with sepsis according to a recent genome-wide expression analysis, among which non-coding RNAs including microRNAs (miRNAs), long non-coding RNAs and circular RNAs are crucial regulators of septic pathogenesis including innate immune response, mitochondrial damage and organ dysfunction (8–10). MicroRNAs widely participate in various physiological processes through regulating the expression of target genes, and new therapies focusing on microRNA intervention have aroused great concern. Recent researches revealed that microRNA intervention alleviated inflammatory response and cell apoptosis in an established kidney or lung injury model under lipopolysaccharide stimulation *in vitro*, indicating that microRNAs were potential therapeutic targets for treatment against sepsis-induced kidney and lung injury (11, 12). MicroRNA-34a (miR-34a) was first discovered as a tumor suppressor in lung cancer through inhibiting tumorigenesis and inducing cell apoptosis (13, 14). Recent evidence has demonstrated that miR-34a promoted reactive oxygen species (ROS) accumulation and cell apoptosis through inhibiting silent information regulator T1 (SIRT1)/nuclear factor erythroid 2-related factor 2 (Nrf2) signaling (15, 16). Additionally, miR-34a suppresses cell autophagy via repressing autophagy related 4B cysteine peptidase (ATG4B) and tumor necrosis factor α (TNF-α) expressions (17, 18). MiR-34a has been reported to mediate lung injuries in multiple lung disorders (19). Moreover, oxidative stress and autophagy play an important role in septic pathogenesis. Thus, we predicted that miR-34a might aggravate sepsis-induced lung injuries through promoting oxidative stress and inhibiting autophagy.

Pyroptosis is also of vital importance for septic pathogenesis, thus we used an established cecal ligation and puncture (CLP) model (20, 21) and investigated the effect of miR-34a on sepsis-induced lung injury via evaluating oxidative stress, inflammatory responses, pyroptosis, and autophagy in septic mice. Our findings tried to clarify the underlying mechanism of miR-34a on regulating lung injury in CLP-induced septic mice.

## Materials and Methods

### Animals

Healthy 8-week-old male C57BL/6 mice were purchased from Liaoning Changsheng biotechnology co., Ltd. (China). Mice were housed in sterilized cages with 45–55% humidity and 12 h light/dark cycles and adaptively fed for 1 week before animal experiment. All animal experiments were carried out following the guideline for the care and use of laboratory animals and approved by the ethics committee of Hainan Medical University (Grant No. 81960346).

### Establishment of CLP Model

Mice were anesthetized by an injection of pentobarbital sodium (50 mg/kg, intraperitoneal injection), followed by creating a 1.5–2 cm incision along the midline of abdominal area to expose cecum. After stripping of mesentery, cecum was ligated at the one-half way from the end of cecum with a number 4 suture except for mice in sham group. Next, cecal puncture was performed at 1 cm from the distal end of ligation by a 21-gauge sterilized needle except for mice in sham group, followed by wound suture. For fluid resuscitation, mice received 1 ml pre-heated normal saline (37°C). For analgesic treatments, mice received buprenorphine (0.05 mg/kg, subcutaneous injection) every 6 h for 2 d.

### Treatments

For the experiments with prophylactic treatment, mice were randomly divided into six groups: sham, sepsis, sepsis+miR-34a antagomir, sepsis+miR-34a agomir, sepsis+NC antagomir, and sepsis+NC agomir. One h before surgery, 20 μg miR-34a antagomir, miR-34a agomir, NC antagomir, and NC agomir (GenePharma, China) were dripped into the airways of mice in sepsis+miR-34a antagomir, sepsis+miR-34a agomir, sepsis+NC antagomir, and sepsis+NC agomir groups, respectively. For the experiments with post-treatment, mice were randomly divided into four groups: sham, sepsis, sepsis+miR-34a antagomir, and sepsis+NC antagomir. Two h after surgery, 20 μg miR-34a antagomir and NC antagomir (GenePharma, China) were dripped into the airways of mice in sepsis+miR-34a antagomir and sepsis+NC antagomir groups, respectively. The *in vivo* transfection reagents of miR-34a antagomir, miR-34a agomir, NC antagomir, and NC agomir were prepared as previously described (22). Meanwhile, an equal dosage of normal saline was dripped in sham and sepsis groups. The survival of mice (10 mice per group) was recorded within 10 days after surgery, and additional agomir/antagomir was supplied at day 6. At 24 h after surgery, lung tissues were collected and fixed for further analysis. Lung wet-to-dry ratio (six mice per group) was also analyzed at 24 h after surgery. For hematoxylin-eosin staining (six mice per group), lung tissues were fixed with 4% paraformaldehyde. For other detection (six mice per group), lung tissues were frozen by liquid nitrogen and then kept at −70°C.

### Hematoxylin-Eosin Staining

Hematoxylin-eosin staining was performed to evaluate the lung injury. Paraffin-embedded lung tissue sections were deparaffinized and stained with hematoxylin for 5 min and eosin for 3 min. Then tissue sections were dehydrated in gradient ethanol and sealed with neutral balsam, followed by observation under a microscope (×200 magnification). The sections were scored as previously described (23).

### Quantitative Real-Time Pcr

Mouse lung tissues were lysed and total RNAs were extracted using Rapid RNA Extraction Kits (Bioteke, China) following the manufacturer's instructions. Then complementary DNA was obtained using M-MLV reverse transcriptase (Takara, Japan) in the presence of oligo (dT)_15_ and random primers, or specific miRNA RT primers. The specific RT primers were listed as below: miR-34a-5p: 5′-GTTGGCTCTGGTGCAGGGTCCGAGGTATTCGCACCAGAGCCAACACAACC-3′; U19: 5′-GTTGGCTCTGGTGCAGGGTCCGAGGTATTCGCACCAGAGCCAACATTGTTTGC-3′. The mRNA levels and miR-34a levels were determined using SYBR Green (Bioteke). U19 and β-actin were used as the internal control, and data were analyzed using the 2^−ΔΔ*CT*^ method. The real-time PCR primers used in this study were listed as below: miR-34a-5p forward primer: 5′-TGGCAGTGTCTTAGCTGGTTGT-3′, reverse primer: 5′-GCAGGGTCCGAGGTATTC-3′; U19 forward primer: 5′-TGTGGAGTTGGTCCTGGTCT-3′, reverse primer: 5′-GTGCAGGGTCCGAGGTATTC-3′; TNF-α forward primer: 5′-CAGGCGGTGCCTATGTCTCA-3′, reverse primer: 5′-GCTCCTCCACTTGGTGGTTT-3′; Interleukin-6 (IL-6) forward primer: 5′-TGTATGAACAACGATGATGCAC-3′, reverse primer: 5′-CTGGCTTTGTCTTTCTTGTT-3′; IL-10 forward primer: 5′-TTAAGGGTTACTTGGGTTGC-3′, reverse primer: 5′-GAGGGTCTTCAGCTTCTCAC-3′; IL-1β forward primer: 5′-CTCAACTGTGAAATGCCACC-3′, reverse primer: 5′-GAGTGATACTGCCTGCCTGA-3′; IL-18 forward primer: 5′-GGCTGCCATGTCAGAAGA-3′, reverse primer: 5′-CCGTATTACTGCGGTTGT-3′; β-actin forward primer: 5′-AATCGTGCGTGACATCAA-3′, reverse primer: 5′-AGAAGGAAGGCTGGAAAA-3′.

### Ros Assay

Mouse lung tissues were cut into pieces of 1 mm^3^ and filtered with 300 mesh nylon net. After washing with PBS buffer, ROS level was evaluated by measuring the fluorescence intensity at the excitation wavelength of 500 nm and the emission wavelength of 525 nm using Reactive oxygen species Assay Kits (Nanjing Jiancheng Bioengineering Institute, China) following the manufacturer's protocols.

### Superoxide Dismutase (Sod) Activity Detection

Mouse lung tissues were homogenized with normal saline (1:9) using a tissue grinder and quantified using BCA Protein Assay Kits (Beyotime, China). The activity of SOD was assessed using SOD assay kits (Nanjing Jiancheng Bioengineering institute) according to the manufacturer's instructions.

### Immunofluorescence Assay

Paraffin-embedded lung tissue sections were deparaffinized and incubated with antigen retrieval solution for 10 min. After washing with PBS buffer for three times, tissue sections were sealed with goat serum for 15 min, incubated with SIRT1 antibody (1:100, Proteintech, China) or ATG4B antibody (1:100, Abcam, UK) overnight at 4°C. Tissue sections were washed with PBS for three times and incubated with Alexa Fluor 594 goat anti-mouse IgG or Alexa Fluor 594 goat anti-rabbit IgG (1:300, Proteintech) for 60 min at room temperature. Then the tissue sections were stained with DAPI (CST, USA) for 5 min and blocked with anti-fluorescence quenching reagent (Solarbio, China), and typical images were captured under a microscope (×400 magnification).

### Western Blot Assay

Mouse lung tissues were smashed using a tissue grinder and lysed with RIPA buffer (Beyotime) to extract total proteins. For nucleoprotein extraction, lung tissues were smashed and extracted using Nuclear Protein Extraction Kit (Beyotime) following the manufacturer's instructions. After quantification using BCA Protein Assay Kits, proteins were separated by SDS-PAGE and transferred to a PVDF membrane (ThermoFisher, USA). Then the PVDF membranes were sealed with 5% BSA and incubated with primary antibodies overnight at 4°C. Primary antibodies used in this study were as follows: SIRT1 antibody (1:1000, CST), Nrf2 antibody (1:500, Proteintech), heme oxygenase-1 (HO-1) antibody (1:1000, Proteintech), IL-1β antibody (1:1000, CST, USA), IL-18 antibody (1:500, Affinity, USA), nucleotide-binding domain and leucine-rich repeat protein-3 (NLRP3) antibody (1:1000, CST), apoptosis-associated speck-like protein containing a CARD (ASC) antibody (1:1000, CST), caspase-1 antibody (1:1000, Abcam, UK), Gasdermin D (GSDMD) antibody (1:1000, CST), LC3 antibody (1:1000, CST), beclin1 antibody (1:1000, CST), p62 antibody (1:1000, CST), ATG9 antibody (1:1000, Proteintech), and ATG4B antibody (1:1000, CST). Histone H3 antibody (1:500, Proteintech) and β-actin antibody (1:2000, Proteintech) were used as the internal control. Corresponding secondary antibodies (1:10000, Proteintech) used in this study were HRP-conjugated goat anti-mouse IgG and HRP-conjugated goat anti-rabbit IgG. After incubation with secondary antibodies at 37°C for 40 min, the protein bands were visualized using ECL reagent (7Sea biotech, China) and were analyzed using Gel-Pro-Analyzer.

### Enzyme-Linked Immunosorbent Assay (Elisa)

Mouse lung tissues were homogenized with normal saline (1:9). After smashing with a tissue grinder, total protein was quantified using BCA Protein Assay Kits (Beyotime). Then the contents of TNF-α, IL-6, IL-10, IL-1β, and IL-18 were determined using commercial ELISA kits (USCN, China).

### Caspase-1 Activity Detection

Mouse lung tissues were homogenized with lysis buffer (1:10). The activity of caspase-1 was detected using caspase-1 activity assay kits (Beyotime). The production of p-nitroaniline (pNA) in samples was used to represent the level of caspase-1 activity.

### Transmission Electron Microscopy

For autophagy observation with electron microscope, lung tissues were fixed with glutaraldehyde. Resin-embedded lung tissue sections were cut at 50–70 nm thickness, stained with uranyl acetate and lead acetate, and then detected under a transmission electron microscope as previously described (24).

### Dual-Luciferase Reporter Assay

HEK293T cells were seeded into a 12-well plate. The luciferase reporter vector containing the wild-type (WT) and mutant (MUT) 3′-untranslated region (3′-UTR) of SIRT1 or ATG4B and miR-34a agomir or negative control (NC) agomir were co-transfected into HEK293T cells. After 48 h, the cells were collected and the relative luciferase activity of cells was measured using Firefly Luciferase Assay Kits (Promega, USA) according to the manufacturer's instructions.

### Statistical Analysis

All statistical analysis was carried out using Graphpad Prism 8.0 (USA). All data were presented as mean ± SD. Results from survival rates were analyzed using Mantel-Cox log-rank test, those from lung injury scores using non-parametric test followed by Mann-Whitney Test, and those from the other data using one-way analysis of variance (ANOVA) followed by Tukey's multiple comparisons test or non-parametric test followed by Dunn's multiple comparisons test. Normality was analyzed using Shapiro-Wilk test and homogeneity of variance using Brown-Forsythe test. And *p* < 0.05 were considered as statistically significant. The *post hoc* power analysis was performed to assess the statistical power of the existing study using the *post-hoc* Power Calculator on a website (https://clincalc.com/Stats~/Power.aspx).

## Results

### Effect of MiR-34a on Survival and Lung Injury in CLP-Induced Septic Mice

**Figure 1:**
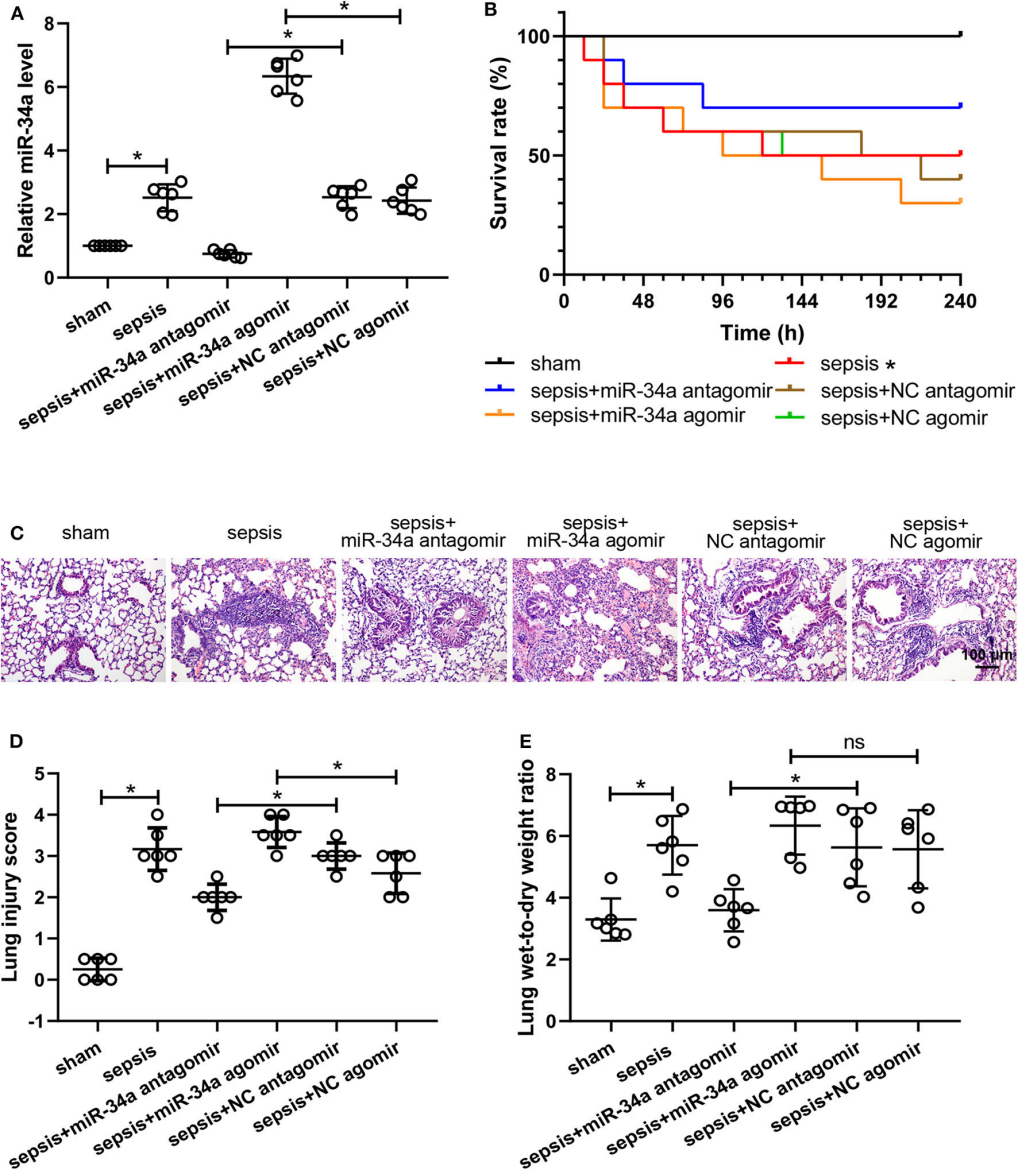
Effect of miR-34a on the survival rate and lung injury in CLP-induced septic mice. C57BL/6 mice were dripped with 20 μg miR-34a antagomir, miR-34a agomir, NC antagomir, or NC agomir at 1 h before surgery and then subjected to CLP to induce sepsis. Lung tissues were collected at 24 h after surgery. **(A)** The relative miR-34a level in mouse lung tissues was measured by quantitative real-time PCR. **(B)** The survival rate of mice in each group after surgery was recorded (*p < 0.05 vs. sham group). **(C,D)** Lung injury was evaluated by Hematoxylin-eosin staining and lung injury score was analyzed (Scale bar = 100 μm). **(E)** The lung wet-to-dry weight ratio was determined at 24 h after surgery *p < 0.05.

The efficiency of miR-34a antagomir and miR-34a agomir was verified by measuring miR-34a levels in lung tissues. The results of quantitative real-time PCR (Figure 1A) showed that miR-34a antagomir significantly down-regulated miR-34a expression in the lung tissues of septic mice (0.70-fold decrease) while miR-34a agomir significantly up-regulated miR-34a level (1.62-fold increase). For survival analysis (Figure 1B), mice in sham group exhibited 100% survival, whereas mice in sepsis group 50% survival. There is no significant difference in the survival rate of mice between sepsis+miR-34a antagomir (70%) and sepsis+NC antagomir (40%) group or sepsis+miR-34a agomir (30%) and sepsis+NC agomir (50%) group. Histological analysis (Figure 1C) revealed that lung tissues in sham group showed a normal structure and those in sepsis, sepsis+NC antagomir, and sepsis+NC agomir groups showed obvious inflammatory cell aggregation and pulmonary interstitial thickening. However, lung tissues in sepsis+miR-34a antagomir group displayed apparently decreased inflammatory cells compared to sepsis+NC antagomir group. On the contrary, lung tissues in sepsis+miR-34a agomir group revealed aggravated pulmonary interstitial thickening and hemocyte infiltration compared to sepsis + NC agomir group. Then the histological changes were reflected by scoring system (Figure 1D). A significant decrease in lung injury scores was shown in sepsis+miR-34a antagomir group (2.00 ± 0.32) compared to those in sepsis+NC antagomir group (3.00 ± 0.32), and A significant increase in lung injury scores was shown in sepsis+miR-34a agomir group (3.58 ± 0.38) compared to those in sepsis+NC agomir group (2.58 ± 0.49). However, compared with sepsis group (3.17 ± 0.52), the lack of significant increase was shown in sepsis+miR-34a agomir group. As shown in Figure 1E, compared with NC antagomir or NC agomir, miR-34a antagomir decreased lung wet-to-dry weight ratio (0.36-fold decrease), whereas miR-34a agomir was lack of a significant increase (0.14-fold increase). All these results suggested that miR-34a inhibition ameliorated lung injury in septic mice.

### Effect of MiR-34a on Oxidative Stress in CLP-Induced Septic Mice

**Figure 2:**
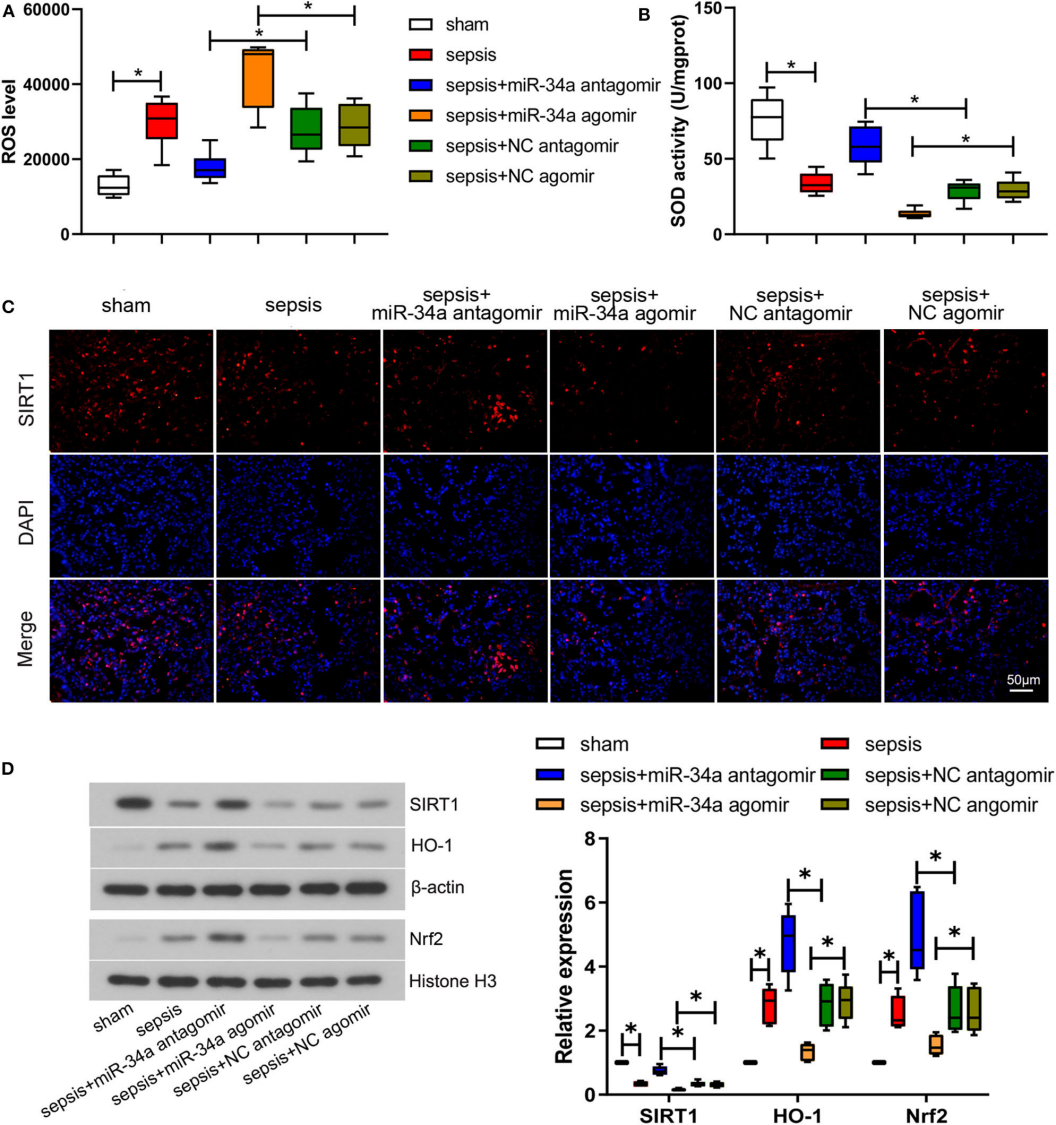
Effect of miR-34a on oxidative stress in CLP-induced septic mice. C57BL/6 mice were dripped with 20 μg miR-34a antagomir, miR-34a agomir, NC antagomir, or NC agomir at 1 h before surgery and then subjected to CLP to induce sepsis. Lung tissues were collected at 24 h after surgery. **(A)** The ROS level in mouse lung tissues was assessed using commercial reactive oxygen species Assay Kits. **(B)** The SOD activity in mouse lung tissues was evaluated using commercial SOD assay kits. **(C)** The expression of SIRT1 was detected using immunofluorescence assay (Scale bar = 50 μm). **(D)** The expression of SIRT1, Nrf2, and HO-1 in mouse lung tissues was determined by western blot assay. Data are presented using box and whisker plots, the line in the box is the median, the upper and lower edge of the box are the 25th and 75th percentiles, and the upper and lower whiskers are the maximum and the minimum *p < 0.05.

The level of oxidative stress was analyzed by determining ROS level (Figure 2A) and SOD activity (Figure 2B) in lung tissues. Compared with NC antagomir or NC agomir, miR-34a antagomir decreased the level of ROS (0.36-fold decrease) in septic mice, while miR-34a agomir increased ROS level (0.50-fold increase). On the contrary, miR-34a antagomir improved the SOD activity (1.03-fold increase) whereas miR-34a agomir inhibited the SOD activity (0.54-fold decrease) in septic mice. The images of SIRT1 expressions in lung tissues (Figure 2C) showed that SIRT1 was mainly located in cell nucleus. MiR-34a antagomir obviously increased SIRT1 expression in lung tissues while miR-34a agomir decreased SIRT1 expression (Figure 2C). As shown in Figure 2D, despite up-regulation of Nrf2 (1.54-fold increase) and HO-1 (1.82-fold increase) expressions in lung tissues, the lack of increase in SIRT1 expression was shown in sepsis group (0.66-fold decrease). MiR-34a antagomir increased SIRT1, Nrf2, and HO-1 expressions (1.24-, 0.86-, and 0.68-fold increase), while miR-34a agomir showed an opposite effect on SIRT1, Nrf2, and HO-1 expressions (0.48-, 0.41-, and 0.54-fold decrease). These results demonstrated that miR-34a inhibition alleviated oxidative stress in septic mice.

### Effect of MiR-34a on Inflammatory Response and Pyroptosis in CLP-Induced Septic Mice

**Figure 3:**
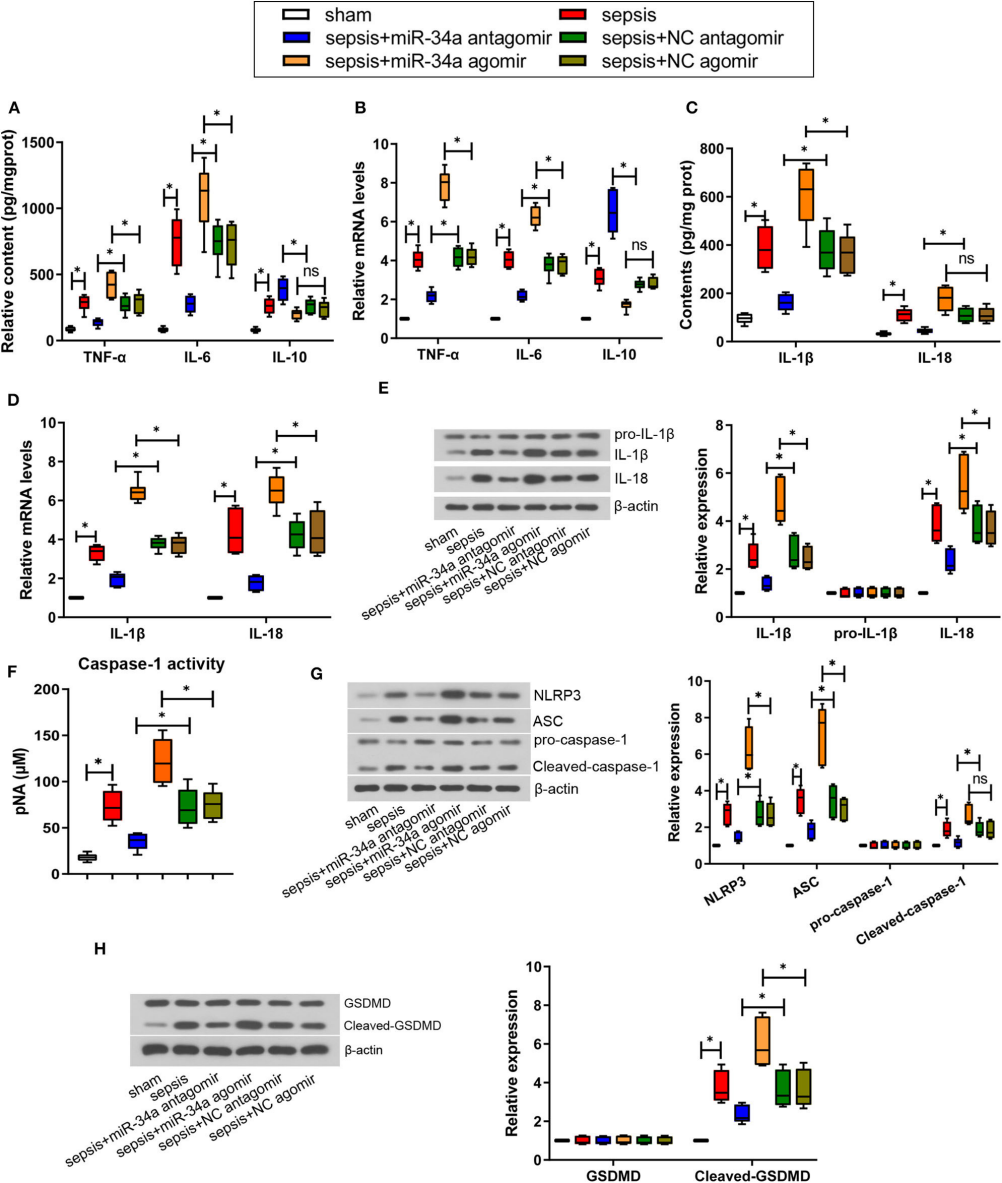
Effect of miR-34a on inflammatory response and pyroptosis in CLP-induced septic mice. C57BL/6 mice were dripped with 20 μg miR-34a antagomir, miR-34a agomir, NC antagomir, or NC agomir at 1 h before surgery and then subjected to CLP to induce sepsis. Lung tissues were collected at 24 h after surgery. **(A)** The content of TNF-α, IL-6, and IL-10 in mouse lung tissues was evaluated using commercial ELISA kits. **(B)** The mRNA level of TNF-α, IL-6, and IL-10 in mouse lung tissues was determined using quantitative real-time PCR. **(C)** The content of IL-1β and IL-18 in mouse lung tissues was evaluated using commercial ELISA kits. **(D)** The mRNA level of IL-1β and IL-18 in mouse lung tissues was determined using quantitative real-time PCR. **(E)** The expression of IL-1β, pro-IL-1β, and IL-18 in mouse lung tissues was evaluated using western blot assay. **(F)** The activity of caspase-1 in mouse lung tissues was determined using a commercial ELISA kit. **(G)** The expression of NLRP3, ASC, pro-caspase-1, and cleaved-caspase-1 in mouse lung tissues was evaluated using western blot assay. **(H)** The expression of GSDMD and cleaved-GSDMD in mouse lung tissues was evaluated using western blot assay. Data are presented using box and whisker plots, the line in the box is the median, the upper and lower edge of the box are the 25th and 75th percentiles, and the upper and lower whiskers are the maximum and the minimum *p < 0.05.

As shown in Figures 3A,C, compared with NC antagomir, miR-34a antagomir significantly reduced the contents of pro-inflammatory factors TNF-α, IL-6, IL-1β, and IL-18 (0.49-, 0.63-, 0.57-, and 0.59-fold decrease), yet increased the anti-inflammatory factors IL-10 contents (0.47-fold increase). On the contrary, compared with NC agomir, miR-34a agomir remarkably increased TNF-α, IL-6, and IL-1β contents (0.45-, 0.49-, and 0.65-fold increase). As shown in Figures 3B,D, compared with NC antagomir, miR-34a antagomir significantly inhibited TNF-α, IL-6, IL-1β, and IL-18 mRNA levels (0.48-, 0.42-, 0.48-, and 0.58-fold decrease) but promoted IL-10 mRNA levels (1.34-fold increase). In comparison with NC agomir, miR-34a agomir remarkably increased TNF-α, IL-6, IL-1β, and IL-18 mRNA levels (0.88-, 0.64-, 0.72-, and 0.51-fold increase). Additionally, IL-1β and IL-18 expressions were significantly down-regulated by miR-34a antagomir (0.48- and 0.39-fold decrease) and up-regulated by miR-34a agomir (0.95- and 0.50-fold increase, Figure 3E). However, both miR-34a antagomir and miR-34a agomir had no significant effect on pro-IL-1β expression. In addition, it was observed that miR-34a antagomir significantly decreased caspase-1 activity (0.51-fold decrease) but miR-34a agomir (0.62-fold increase) exhibited an opposite effect (Figure 3F). Then NLRP3 inflammasome-related proteins and pyroptosis-related proteins were detected. MiR-34a antagomir down-regulated the expression of NLRP3, ASC, cleaved caspase-1, and cleaved-GSDMD (0.48-, 0.47-, 0.38-, and 0.36-fold decrease), while miR-34a agomir up-regulated the expression of NLRP3, ASC, and cleaved-GSDMD (1.34-, 1.36-, and 0.66-fold increase, Figures 3G,H). Nevertheless, miR-34a antagomir and miR-34a agomir had no significant effect on pro-caspase-1 and GSDMD expression, and miR-34a agomir had no significant effect on cleaved-caspase-1 expression (Figures 3G,H). These findings indicated that miR-34a inhibition ameliorated inflammatory response and pyroptosis in septic mice.

### Effect of MiR-34a on Autophagy in CLP-Induced Septic Mice

**Figure 4:**
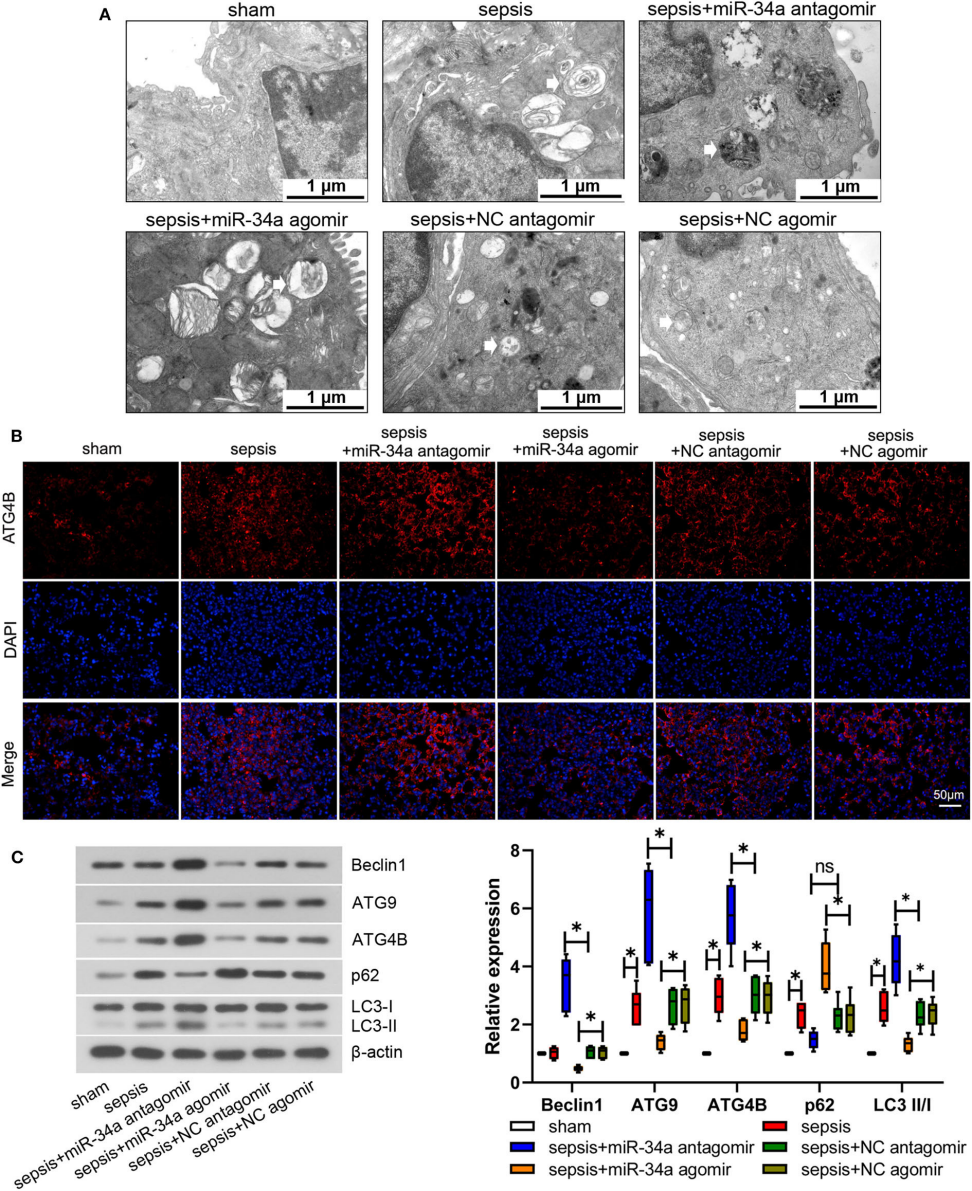
Effect of miR-34a on autophagy in CLP-induced septic mice. C57BL/6 mice were dripped with 20 μg miR-34a antagomir, miR-34a agomir, NC antagomir, or NC agomir at 1 h before surgery and then subjected to CLP to induce sepsis. Lung tissues were collected at 24 h after surgery. **(A)** Autophagy of mouse lung tissues was observed under an electron microscope (Scale bar = 1 μm). **(B)** The expression of ATG4B in mouse lung tissues was detected using immunofluorescence (Scale bar = 50 μm). **(C)** The expression of beclin1, ATG9, ATG4B, p62, and LC3 II/I was determined using western blot assay. Data are presented using box and whisker plots, the line in the box is the median, the upper and lower edge of the box are the 25th and 75th percentiles, and the upper and lower whiskers are the maximum and the minimum *p < 0.05.

Mouse lung tissues were observed under an electron microscope to evaluate autophagy. No obvious autophagy was observed in sham group (Figure 4A). Typical autophagosomes were observed in mouse lung tissues in sepsis, sepsis+miR-34a agomir, sepsis+NC agomir, and sepsis+NC antagomir groups. Of note, typical autolysosomes were observed in sepsis+miR-34a antagomir group (Figure 4A). As shown in Figure 4B, ATG4B protein expression was elevated in sepsis+miR-34a antagomir group but decreased in sepsis+miR-34a agomir group compared with that in sepsis+NC antagomir group or sepsis+NC agomir group. In addition, miR-34a antagomir increased beclin1, ATG9, ATG4B, and LC3 II/I expressions (2.27-, 1.23-, 0.90-, and 0.82-fold increase) in mouse lung tissues, while miR-34a agomir inhibited beclin1, ATG9, ATG4B, and LC3 II/I expressions (0.54-, 0.48-, 0.39-, and 0.44-fold decrease) and promoted p62 expression (0.72-fold increase, Figure 4C). These findings manifested that miR-34a inhibition facilitated autophagy in septic mice.

### MiR-34a Targets Sirt1 and Atg4B

**Figure 5:**
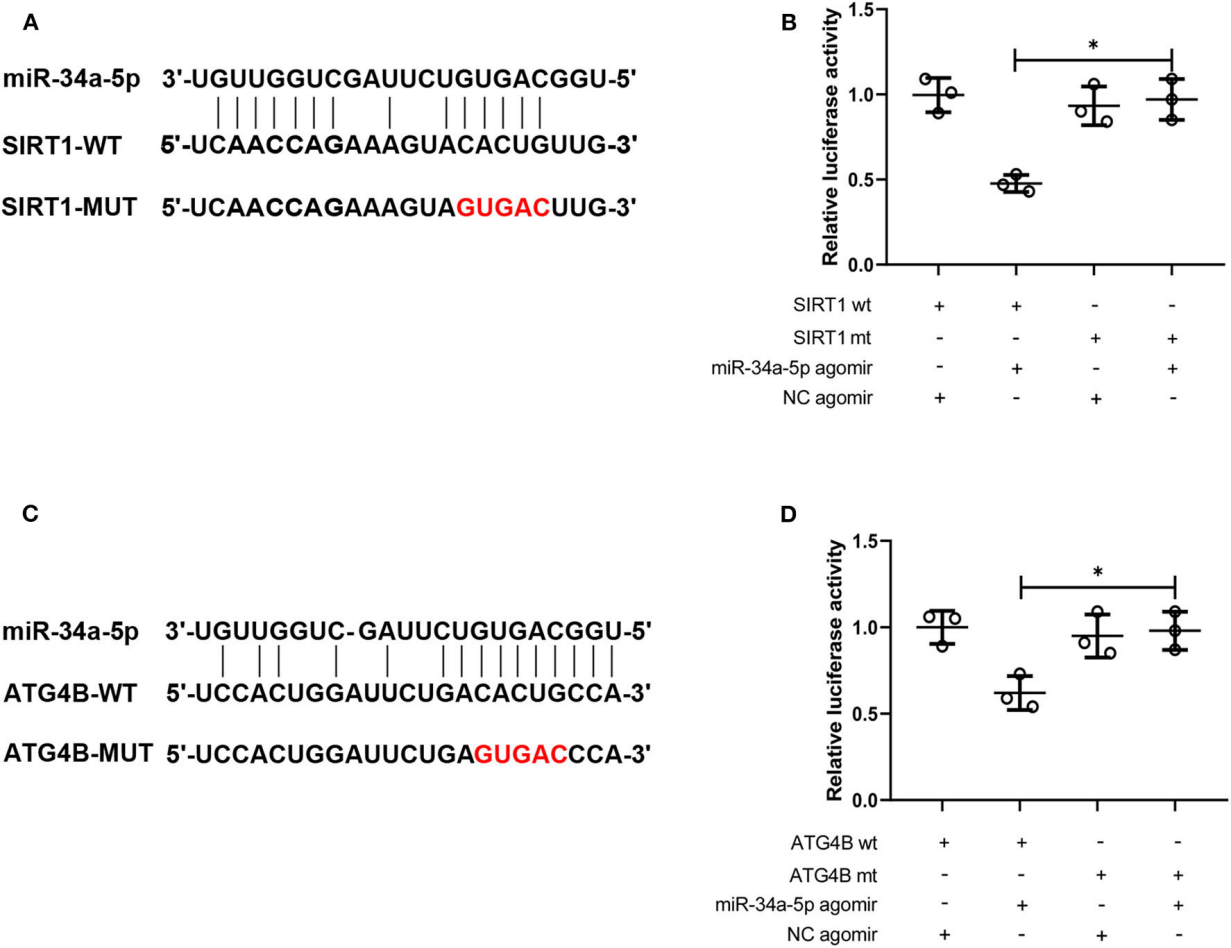
MiR-34a targets SIRT1 and ATG4B. **(A)** The binding site of miR-34a on SIRT1 3′-UTR was displayed. **(B)** The interaction between miR-34a and SIRT1 was validated using dual-luciferase reporter assay. **(C)** The binding site of miR-34a on ATG4B 3′-UTR was displayed. **(D)** The interaction between miR-34a and ATG4B was validated using dual-luciferase reporter assay *p < 0.05.

The binding between SIRT1 3′-UTR and miR-34a or ATG4B 3′-UTR and miR-34a was predicted and the binding sites were shown in Figures 5A,C. Then the interaction between miR-34a and SIRT1 or miR-34a and ATG4B was validated. The luciferase activity in SIRT1-WT+miR-34a-5p agomir group (0.48 ± 0.05) was notably lower than that in SIRT1-MUT+miR-34a-5p agomir group (0.97 ± 0.12, Figure 5B). Similar results were found in ATG4B-WT+miR-34a-5p agomir group (0.62 ± 0.10) and ATG4B-MUT+miR-34a-5p agomir group (0.98 ± 0.11, Figure 5D). These results demonstrated that SIRT1 and ATG4B could serve as a downstream target of miR-34a.

### Post-treatment MiR-34a Inhibition Ameliorates CLP-Induced Lung Injury, Oxidative Stress, Inflammatory Response, Pyroptosis, and Autophagy in Septic Mice

**Figure 6:**
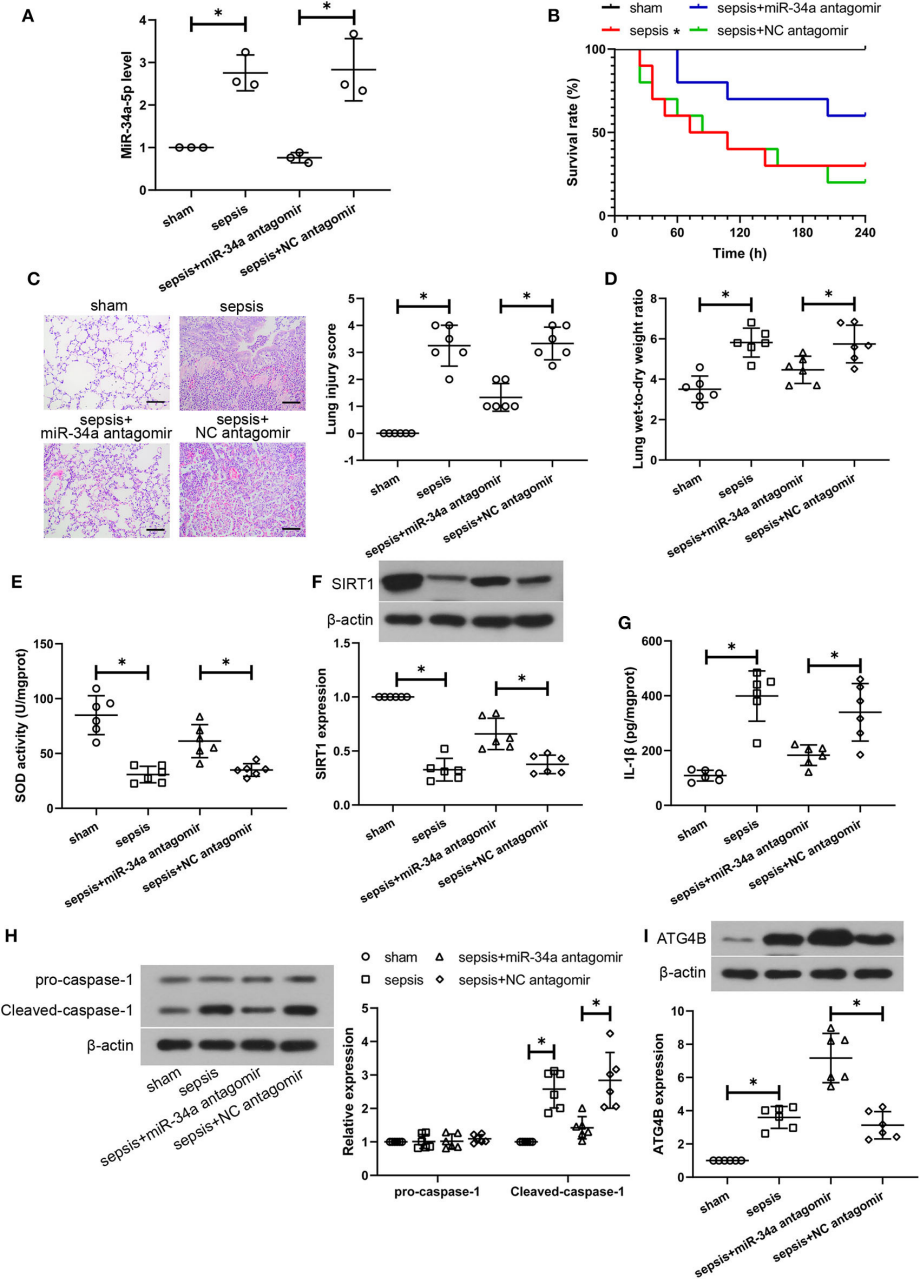
Post-treatment with miR-34a antagomir alleviates sepsis-induced lung injuries. C57BL/6 mice were subjected to CLP to induce sepsis and then dripped with 20 μg miR-34a antagomir or NC antagomir at 2 h after surgery. Lung tissues were collected at 24 h after surgery. **(A)** The relative miR-34a level in mouse lung tissues using quantitative real-time PCR. **(B)** The survival rate of mice was recorded (*p < 0.05 vs. sham group). **(C)** Lung injury analysis using Hematoxylin-eosin staining (Scale bar = 100 μm). **(D)**The lung wet-to-dry weight ratio was determined at 24 h after surgery. **(E)** The activity of SOD using a commercial assay kit. **(F)** SIRT1 expression using western blot assay. **(G)** The content of IL-1β using ELISA. **(H)** Cleaved-caspase-1 and pro-caspase-1 expression using western blot assay. **(I)** ATG4B expression using western blot assay *p < 0.05.

To further verify the therapeutic effect of miR-34a inhibition on CLP-induced lung injury, some parts of the study were repeated with a post-treatment of miR-34a antagomir. As shown in Figure 6A, miR-34a antagomir significantly down-regulated miR-34a expression in the lung tissues of septic mice (0.73-fold decrease, power: 100.0%). However, miR-34a antagomir was lack of a significant improvement in the survival of septic mice (power: 44.2%, Figure 6B). Lung tissues in sepsis+miR-34a antagomir group (Figure 6C) revealed mitigated pulmonary interstitial thickening and hemocyte infiltration compared to sepsis+NC antagomir group. The scoring of histological changes (Figure 6C) showed that a significant decrease in lung injury scores was shown in sepsis+miR-34a antagomir group (0.60-fold decrease, power: 100.0%) compared to those in sepsis+NC antagomir group. Additionally, miR-34a antagomir significantly decreased lung wet-to-dry weight ratio (0.22-fold decrease, power: 78.1%, Figure 6D). As shown in Figures 6E,F, miR-34a antagomir significantly promoted SOD activity (0.75-fold increase, power: 97.9%) and SIRT1 expression (0.75-fold increase, power: 97.5%) in lung tissues. However, miR-34a antagomir significantly decreased IL-1β content (0.46-fold decrease, power: 93.1%, Figure 6G) and inhibited cleaved-caspase-1 expression (0.50-fold decrease, power: 97.3%, Figure 6H) in lung tissues. As shown in Figure 6I, miR-34a antagomir significantly enhanced ATG4B expression (1.29-fold increase, power: 100.0%) in lung tissues. These results implied that post-treatment with miR-34a antagomir was effective in alleviating CLP-induced lung injury, oxidative stress, inflammatory response, pyroptosis, and autophagy in septic mice.

## Discussion

The latest guideline for sepsis management indicates that early diagnosis is critical for sepsis treatment (25). MicroRNAs have been shown as a potential biomarker for the early diagnosis of multiple diseases including cancer, neurodegenerative diseases, and cardiovascular disease (26–29). Currently, several mechanisms which contribute to sepsis pathogenesis, including inflammation, autophagy, and pyroptosis, have been demonstrated to be regulated by microRNAs (10). In the present study, we investigated the effect of miR-34a on lung injury, oxidative stress, inflammation, pyroptosis, and autophagy in CLP-induced sepsis mouse model using commercial miR-34 antagomir/agomir. CLP is commonly used to establish murine sepsis models as it closely simulates the progression of human sepsis. CLP induces polymicrobial infection in abdominal cavity and further triggers systemic inflammation, and eventually results in organ dysfunction (30, 31). Lung injury is one of the most frequent organ dysfunctions in sepsis (32). We found that miR-34a inhibition exhibited a tendency to improve the survival of septic mice, accompanied with significantly alleviated lung injury as evidenced by decreased inflammatory cells and water content of lung. On the contrary, miR-34a overexpression exhibited a tendency to decrease the survival rate of septic mice and aggravate lung injuries. These results implied that miR-34a played an important role in sepsis-induced lung injuries.

Growing evidence has suggested that oxidative stress which is induced by ROS accumulation and reduced activity of antioxidant enzymes plays a non-negligible role in the progress of sepsis as it leads to mitochondrial dysfunction and further organ failure (33). In our study, CLP triggered ROS accumulation and inhibited the activity of SOD, indicating that elevated levels of oxidative stress were existed in septic mice. However, down-regulation of miR-34a expression suppressed oxidative stress and up-regulation of miR-34a expression exhibited an opposite effect, indicating that miR-34a aggravated oxidative stress in the development of sepsis. The transcription factor Nrf2 has been identified as a positive antioxidant regulator and the activation of Nrf2 signaling can prevent oxidative stress-induced injury in cells and tissues (34). The Nrf2-regulated downstream factor HO-1 has been confirmed as a potent antioxidant, and up-regulation of HO-1 is able to inhibit mitochondrial oxidative stress (35). Additionally, Nrf2 is a downstream factor of SIRT1. SIRT1 has been reported to suppress oxidative stress via regulating SOD activity in vascular endothelial cells (36). Of note, miR-34a inhibition reduced ROS production via targeting SIRT1 in mice (16, 37). In the present study, miR-34a overexpression inhibited SIRT1 expression in lung tissues and miR-34a could directly bind to the 3′-UTR of SIRT1. Moreover, miR-34a overexpression inhibited HO-1 and nuclear Nrf2 expressions in lung tissues. Taken together, it was concluded that miR-34a might aggravate oxidative stress via targeting SIRT1 and inhibiting Nrf-2/HO-1 pathway.

Despite the updating diagnostic criteria, inflammation remains the fundamental pathogenesis, and essential therapeutic target for sepsis, and excessive inflammatory responses are responsible for early deaths in patients with sepsis (6, 38). In the present study, we found that miR-34a inhibition alleviated CLP-induced inflammatory responses as evidenced by decreased contents and mRNA levels of pro-inflammatory cytokines TNF-α and IL-6, and correspondingly increased contents and mRNA levels of anti-inflammatory cytokine IL-10. Therefore, our findings indicated that miR-34a inhibition ameliorated inflammation in septic mice. Recent studies have shown that pyroptosis plays a pivotal role in lung injury (39, 40). Pyroptosis is known as a form of pro-inflammatory cell death program, and this process is caspase-1-dependent. The NLRP3 inflammasome complex composed of NLRP3, ASC, and procaspase-1 cleaves procaspase-1 for caspase-1 activation (cleaved-caspase-1), and results in the processing and maturation of IL-1β and IL-18 that can induce pyroptosis. The induction of pyroptosis further promotes the secretion of IL-1β and IL-18. GSDMD mediates pore formation in cells undergoing pyroptosis (41). GSDMD can be cloven by activated caspase-1 and this cleavage is required for the release of IL-1β and IL-18 and pyroptosis (42). In the present study, miR-34a inhibition promoted NLRP3 and ASC expressions, caspase-1 cleavage and activation, the secretion and expression of IL-1β and IL-18, and GSDMD cleavage in CLP-induced septic mice, indicating that miR-34a enhanced pyroptosis in CLP-induced septic mice. It is first reported that miR-34a is involved in pyroptosis.

Autophagy activation has been reported to improve sepsis-induced lung injury (43). Increased oxidative stress triggers the up-regulation of pyroptosis and the down-regulation of autophagy. Beclin1, ATG9, and ATG4B are critical proteins for the induction of autophagy. LC3 conversion (LC3-I to LC3-II) and the degradation of p62 are indicators of autophagy (44). MiR-34a has been reported to inhibit autophagy in retinoblastoma cells (45). In the present study, we found that miR-34a inhibition induced autophagy as evidenced by up-regulation of ATG9, ATG4B, and beclin1 expression, accompanied with the accumulation of LC3-II. In addition, miR-34a could bind to the 3′-UTR of ATG4B. These results indicated that miR-34a might directly regulate autophagy via targeting ATG4B and indirectly inhibit autophagy by elevated oxidative stress.

Our study has several limitation. First, despite alleviation of sepsis-induced lung injury, miR-34a inhibition only had a small effect on the survival rate of septic mice. Second, we found that miR-34a regulated SIRT1 and ATG4B expressions, oxidative stress and autophagy in lung tissues. Furthermore, miR-34a could bind to SIRT1 and ATG4B. However, it was uncertain whether miR-34a regulated oxidative stress and autophagy by targeting SIRT1 and ATG4B. The further validation will be needed *in vivo* in our future study. Additionally, the *post-hoc* power analysis exhibited a power of 44.2% in the survival analysis and a power of 78.1% in the lung wet-to-dry weight ratio analysis. Generally, an appropriate power should be more than 80% (46). Hence, the small sample size presented in the survival analysis and lung wet-to-dry weight ratio analysis is another limitation of this study.

In conclusion, our results demonstrated that miR-34a inhibition alleviated lung injury in septic mice through suppressing oxidative stress, inflammatory response, and pyroptosis, and activating autophagy. However, miR-34a up-regulation exhibited an opposite effect on oxidative stress, inflammatory response, pyroptosis, and autophagy in septic mice. These results indicated that miR-34a was implicated in the regulation of oxidative stress, inflammatory response, pyroptosis, and autophagy in septic mice, which might be related to sepsis-induced lung injury. Moreover, miR-34a can serve as a potential therapeutic target for treatment against sepsis-induced lung injury.

## Data Availability Statement

The raw data supporting the conclusions of this article will be made available by the authors, without undue reservation.

## Ethics Statement

This animal study was reviewed and approved by ethics committee of Hainan Medical University.

## Author Contributions

SC, CL, and RD conceived and designed the experiments. SC, ZH, XY, FX, and WZ performed experiments. SC and SY analyzed the data. SC wrote the manuscript. All authors read and approved the final manuscript.

## Conflict of Interest

The authors declare that the research was conducted in the absence of any commercial or financial relationships that could be construed as a potential conflict of interest.
